# Comparing NGS-Based identification of bloodstream infections to traditional culture methods for enhanced ICU care: a comprehensive study

**DOI:** 10.3389/fcimb.2024.1454549

**Published:** 2024-09-12

**Authors:** Wei Wang, Varun Chauhan, Yutian Luo, Sonu Sharma, Chenxi Li, Huaisheng Chen

**Affiliations:** ^1^ Department of Endocrinology, Shenzhen People’s Hospital, Second Clinical Medical College of Jinan University, First Affiliated Hospital of Southern University of Science and Technology, Shenzhen, Guangdong, China; ^2^ Department of Microbiology, Faculty of Applied Sciences and Biotechnology, Shoolini University, Solan, India; ^3^ Department of Critical Care Medicine, Shenzhen People’s Hospital, Second Clinical Medical College of Jinan University, First Affiliated Hospital of Southern University of Science and Technology, Shenzhen, Guangdong, China; ^4^ Department of Pharmacy, DIT University, Mussoorie, Uttarakhand, India

**Keywords:** NGS-based identification in bloodstream infection next-generation sequencing (NGS), bloodstream infections (BSIs), molecular diagnostics, ICU pathogen identification, Bactfast and Fungifast

## Abstract

**Background:**

Accurate identification of infectious diseases using molecular techniques, such as PCR and NGS, is well-established. This study aims to assess the utility of Bactfast and Fungifast in diagnosing bloodstream infections in ICU settings, comparing them against traditional culture methods. The objectives include evaluating sensitivity and specificity and identifying a wide range of pathogens, including non-culturable species.

**Methods:**

We collected 500 non-duplicate blood samples from ICU patients between May 2019 and May 2020. Specimens underwent traditional culture, MALDI-TOF, VITEK^®^2 compact system, and NGS-based Bactfast and Fungifast analyses.

**Results:**

Out of the 500 samples, 26.8% (n=134) showed bacterial growth via traditional culture methods, while 4.8% (n=24) were positive for fungal growth. MALDI-TOF and VITEK^®^2 compact system yielded comparable results, identifying 26.4% (n=132) of specimens with bacterial growth. NGS-based Bactfast detected bacterial presence in 38.2% (n=191) of samples, including non-culturable bacteria missed by traditional methods. However, NGS-based Fungifast showed concordant fungal detection rates with culture methods. Among identified pathogens by culture method included *Klebsiella pneumoniae* 20.89% (n=28), *Enterococcus faecalis* 18.65% (n=25), *Escherichia coli* 15.67% (n=21), *Pseudomonas aeruginosa* 12.68% (n=17), *Acinetobacter baumannii* 10.44% (n=14), various *Streptococcus* species 7.46% (n=10), *Mycobacterium tuberculosis* 6.71% (n=9), *Mycobacterium abscessus* 4.47% (n=6), and *Salmonella spp* 2.98% (n=4). Non-culture-based NGS identified additional (n=33) pathogens, including *Klebsiella pneumoniae* 27.27% (n=9), *Bacteroides fragilis* 21.21% (n=7), *Aerococcus viridans* 15.15% (n=5), *Elizabethkingia anopheles* 12.12% (n=4), *Aeromonas salmonicida* 9% (n=3), *Clostridium* 9% (n=3), and *Bacteroides vulgatus* 6% (n=2). *Candida albicans* was reported in 5% (n=24) of samples by both methods.

**Conclusion:**

NGS-based Bactfast and Fungifast demonstrate high sensitivity in identifying a wide array of bacterial and fungal pathogens in ICU patients, outperforming traditional culture methods in detecting non-culturable organisms. These molecular assays offer rapid and comprehensive diagnostic capabilities, potentially improving clinical outcomes through timely and accurate pathogen identification.

## Introduction

Bloodstream infections (BSIs) are a significant concern in intensive care unit (ICU) settings, contributing to high morbidity and 60% mortality rates (Bassetti et al., 2016). Accurate and timely identification of the causative pathogens is crucial for effective treatment and improved patient outcomes (Caliendo et al., 2013; Munro et al., 2024). Conventional techniques for identifying pathogens from clinical samples include culture-based tests followed by biochemical assays and susceptibility testing (Kabiraz et al., 2023). However, these methods have limitations, such as inconclusive results due to prior antibiotic therapy, the fastidious nature of some organisms, or the inherent limitations of culture as a diagnostic tool (Zhang et al., 2021). Furthermore, culture-based methods are often associated with significant delays, and while advancements like MALDI-TOF have facilitated bacterial identification, these methods remain culture-dependent (Li et al., 2022). The challenge of culture-based methods is even more pronounced for fungal detection (Otašević et al., 2018). Fungal cultures typically require extended incubation periods of up to four weeks to identify slow-growing species, impacting the clinical utility of these tests due to impractical delays in results (Bosshard, 2011). To address these limitations, molecular diagnostics have gained traction in clinical microbiology for pathogen identification. Molecular techniques, such as PCR and RT-PCR, provide rapid results by detecting the DNA or RNA of pathogens directly from clinical samples (Yang and Rothman, 2004; Anahtar et al., 2020). These tools have significantly improved the diagnosis of various infections with greater accuracy and speed, which is particularly important in ICU settings where prompt and precise diagnoses are vital for effective treatment. Metagenomics, the study and characterization of microorganisms via nucleic acid sequences, has greatly benefited from the development of next-generation sequencing (NGS) (Chen et al., 2022). NGS enables the detection of a wide range of pathogens, including those that are non-culturable, by analyzing ribosomal RNA regions such as 16S rRNA for bacteria and internal transcribed spacer (ITS) regions for fungi (Satam et al., 2023). This technology allows for the comprehensive assessment of microbial communities within a sample, providing detailed insights into the presence of individual species. The application of NGS in clinical laboratories has become increasingly widespread, enhancing the diagnostics of infectious diseases, immune disorders, and hereditary conditions. NGS-based metagenomics offers a culture-independent approach to pathogen detection, generating high-throughput sequence data that can be analyzed to identify microbial content within a specimen (Tang et al., 2021). This method significantly improves the speed and accuracy of pathogen identification, making it a valuable tool for enhancing ICU care. Bactfast and Fungifast are NGS-based diagnostic tests developed by Credence Genomics, designed to provide rapid and accurate identification of bacterial and fungal pathogens from clinical samples. These assays use customized primers to amplify partial 16S rRNA and ITS regions, allowing for the multiplexing of multiple samples in a single sequencing run (Ramanathan et al., 2023). The amplified gene regions are purified, sequenced, and analysed using proprietary bioinformatics pipelines to identify the organisms within the sample. This study aims to evaluate the performance of NGS-based diagnostics compared to traditional culture methods, MALDI-TOF, and VITEK^®^2 compact systems in the context of ICU bloodstream infections. By addressing the current challenges and demonstrating the advantages of NGS, we aim to provide a comprehensive overview of its potential impact on ICU diagnostics and patient outcomes. BSIs are critical and common in ICU patients, often leading to severe complications. Moreover, they provide a relatively homogeneous sample type for studying pathogen profiles and resistance patterns. Our objectives include assessing the sensitivity, specificity, and overall utility of these NGS-based tools in diagnosing BSIs, with a focus on their ability to detect a broad range of pathogens, including non-culturable species. By exploring the potential impact of these advanced diagnostics on clinical decision-making and patient outcomes, we hope to demonstrate their value in improving the management of BSIs in critically ill patients.

## Methods

This study was conducted per the guidelines of the Declaration of Helsinki and approved by the Institutional Ethics Committee of the Shoolini University with reference number: SUBMS-2022-EC-025 which complies with international ethical standards. All experiments were compliant with the institutional biosafety regulations.

### Study design and sample collection

This is the single centre study conducted between May 2019 to May 2020 at Department of the Microbiology, in Shoolini University. A total of 500 non-duplicate blood specimens were collected based on the physician. Each sample was collected from a single patient to ensure unique data points and avoid duplication. The physician orders the blood culture as a suspected infection. Inclusion criteria comprised adult ICU patients with suspected bloodstream infections, while exclusion criteria included patients with recent antibiotic treatment and those with chronic infections or immune-compromised conditions. The study included patients with various underlying health conditions such as sepsis, multiple organ failure, and chronic diseases. The samples were collected within the first 48 hours of ICU admission. This timing helps differentiate between community-acquired and nosocomial infections. The 10 ml blood samples were collected in the sterile condition in an added EDTA vial. After the initiating collection of the samples, each 2 ml sample was sent to the NGS laboratory, followed by microbiology laboratory and Biophysical instrument lab for MALDI-TOF. All the time 4°c temperature mainlined.

### Identification of pathogens

Upon receipt at the microbiology laboratory, blood samples were processed using traditional culture methods to isolate and identify the causative pathogens (Plato et al., 2019). Pathogen identification was performed using several biochemical tests, including Gram staining, catalase, oxidase tests, and API 20E for Enterobacteriaceae identification. No antibiotics were used during plating. Enriched cultures were streaked on blood agar and MacConkey agar to isolate individual colonies. Each blood sample was inoculated into aerobic and anaerobic blood culture bottles containing an enrichment broth to endorse microbial growth. The bottles were then incubated at 37°C using an automated blood culture system BACTEC FX (Becton Dickinson, Franklin Lakes, NJ, USA). The blood culture bottles were monitored continuously for up to 5 days for microbial growth, indicated by changes in the colour of the broth, which indicate the production of CO_2_ by metabolizing bacteria (Yarbrough et al., 2021). Enriched culture followed by the streak on the blood agar and MacConkey agar to isolate individual colonies. These plates were incubated at 37°C for 24 to 48 hours. After the isolated pure colonies were subjected to Gram’s staining and examined microscopically to determine their Gram reaction (positive or negative) and morphology (cocci or bacilli). This initial characterization guided the selection of further biochemical tests. Isolated bacterial colonies were picked and suspended in a 0.85% saline solution to prepare a standardized inoculum. This suspension was loaded into VITEK^®^2 compact system ID-N935 cards with different bacterial species. The VITEK^®^2 compact system automates the incubation, reading, and interpretation of the results as previously described (Patil et al., 2019). comparing the biochemical reactions to a comprehensive database. In parallel with traditional culture methods, 2 ml of each blood sample was processed for MALDI-TOF MS analysis. After positive cultures were confirmed, bacterial colonies were directly transferred onto a MALDI target plate using a sterile loop. A matrix solution- α-cyano-4-hydroxycinnamic acid (CHCA) dissolved in an organic solvent, was applied over the bacterial sample on the target plate. The matrix co-crystallizes with the sample and facilitates ionization. The prepared target plate was then inserted into the Bruker MALDI Biotyper system (Bruker Daltonics, Bremen, Germany). The system uses a laser to ionize the sample-matrix mixture, producing characteristic mass spectra based on the protein composition of the bacteria. The generated spectral data were analysed by the Biotyper software, which compares the spectra against a reference database containing profiles of known organisms (Tsuchida et al., 2020). The software provided a confidence score indicating the reliability of the identification. We positive control use ATCC25922 while deionised water was used as the negative control.

### NGS based diagnosis

Each blood samples were anonymized and assigned unique sample IDs for processing. Each sample underwent a standardized preprocessing protocol tailored for Bactfast and Fungifast analyses (BactFast, n.d). The process began with DNA extraction using the QIAamp DNA Mini Kit (Qiagen), following the manufacturer’s instructions to ensure a consistent elution volume of 50μL. Rigorous validation and quality control measures were implemented for the NGS assays. These included the use of internal controls, ensuring sufficient sequencing depth, and comprehensive data quality checks to verify the accuracy and reliability of the sequencing results. To monitor potential contamination, negative extraction controls (EC) were included in each batch, which typically comprised 5 to 10 samples, depending on the collection volume. All extraction procedures were conducted within a Level II Biosafety Cabinet under controlled conditions at 4°C. Following DNA extraction, library preparation commenced with customized protocols specific to Bactfast and Fungifast assays, as previously described (Paton et al., 2021). Amplified DNA products were electrophoresed on 1.5% agarose gels alongside a 50 bp DNA ladder, negative extraction controls (EC), non-template controls (NTC), and positive controls. Gel visualization under UV transillumination using ethidium bromide confirmed successful amplification. Positive amplicons underwent purification using 1X NucleoMagR size select beads (Macherey-Nagel) as per the manufacturer’s instructions, followed by elution in a low TE buffer. Quantification of purified libraries was performed using the Qubit dsDNA HS Assay Kit (Invitrogen). Subsequently, template preparation for sequencing was carried out using the Ion Torrent S5 XL system with the OneTouch 2 template preparation protocol. Samples were pooled in batches of either 20 or 60 for sequencing on the Ion 520 or 530 chips, respectively, following the manufacturer’s guidelines for 400 bp sequencing runs. Raw sequencing data were collected and subjected to a proprietary bioinformatics pipeline dedicated to species identification. Bioinformatics analysis involved initial processing of unaligned BAM (UBAM) files, filtering reads with a quality score greater than Q20. Each file was named according to sample ID, barcode, and sample type for systematic organization. Trimmed reads underwent stringent quality control checks, including Phred Quality Score cutoffs and minimum read length thresholds. Expected read numbers per sample ranged from 75,000 to 100,000, ensuring robust sequencing depth for reliable analysis. Bactfast v2.2 and Fungifast 1.1 protocols guided sequence alignment and species identification, employing two parallel pipelines for validation. The primary pipeline utilized MegaBLAST (blast-2.5.0) for alignment against NCBI databases, while a modified MySQL application integrated taxonomy and homology data to construct phylogenetic trees based on accession numbers. Simultaneously, the kraken2 (version 2) pipeline linked to NCBI RefSeq data enabled comparative analysis against a curated reference library. This library included singular rRNA and 16S rRNA positions for each organism, ensuring comprehensive species resolution. Both pipelines were validated against known datasets to confirm accuracy and specificity. Post-analysis, results were categorized into MySQL summary files detailing taxonomic classifications and Kraken summary files representing hierarchical alignments against Refseq version 91 (Wood et al., 2019, p. 2). Results were reported based on clinical parameters, site of infection, symptoms, and pathogen abundance by medical microbiologists. Contaminant control measures included regular use of EC and NTC in extraction and PCR runs to mitigate environmental influences. Additionally, environmental metagenomic profiling of ICU settings provided baseline data to enhance bioinformatic analysis by filtering environmental flora abundance. Expert review panels comprising infectious disease specialists, physicians, and medical microbiologists evaluated NGS and culture results for clinical significance. Discordant results between methods were categorized based on concordance with clinical history and epidemiology, ensuring accurate interpretation of findings to guide patient management strategies. This comprehensive approach in the NGS-based Bactfast and Fungifast assays underscored their utility in enhancing pathogen detection and characterization in ICU patients, surpassing conventional culture methods in sensitivity and rapidity of diagnostic outcomes.

### Statistical analysis

Statistical analyses were performed using R version 4.0.3 (R Core Team, 2020). Categorical variables such as gender and clinical conditions were compared using the Chi-square test to assess differences between groups. A *p*-value < 0.05 was considered statistically significant.

## Results

### Study population

A total of 500 unique blood specimens were collected from ICU patients between January 2023 and December 2023 at the Department of Microbiology. The study population comprised 56.4% (n=282) males and 43.6% (n=218) females (43.6%) with a mean age of 58.2 years (SD ± 12.4 years) and an interquartile range (IQR) of 49 to 68 years. The study included adult patients aged 18 and above. Patients included in this study were admitted to the ICU for various conditions, including sepsis 45% (n=225), pneumonia 20% (n=100), urinary tract infections 15% (n=75), post-surgical infections (10%) (n=50), and other critical conditions such as severe trauma and pancreatitis (n=50%) (**Table 1**). All patients were suspected of having bloodstream infections, warranting blood culture orders by attending physicians.

**Table 1:** Demographic and clinical characteristics of study population.

Characteristic	Number (n)	Percentage %	P value
Total Specimens (n=500)			
**Gender**			<0.05
Male	282	56.4	
Female	218	43.6	
Age			
Mean ± SD	58.2 ± 12.4		
Interquartile Range	49 - 68		
**Clinical conditions**			<0.05
Sepsis	225	45	
Pneumonia	100	20	
Urinary tract infections	75	15	
Post-surgical infections	50	10	
Other critical conditions*	50	10	

*Includes severe trauma and pancreatitis.

### Sample collection and transport

Out of the 500 samples collected, 498 samples (99.6%) were successfully transported and processed without any significant delays or deviations from protocol. Each 10 ml blood sample was collected under sterile conditions and added to EDTA vials to prevent clotting. Following collection, 2 ml aliquots of each sample were sent to three different laboratories: the NGS laboratory for molecular analysis, the microbiology laboratory for traditional culture methods, and the biophysical instrument lab for MALDI-TOF analysis. The transport and storage of all samples maintained a consistent temperature of 4°C to preserve sample integrity.

### Species identification-traditional

**Figure 1:**
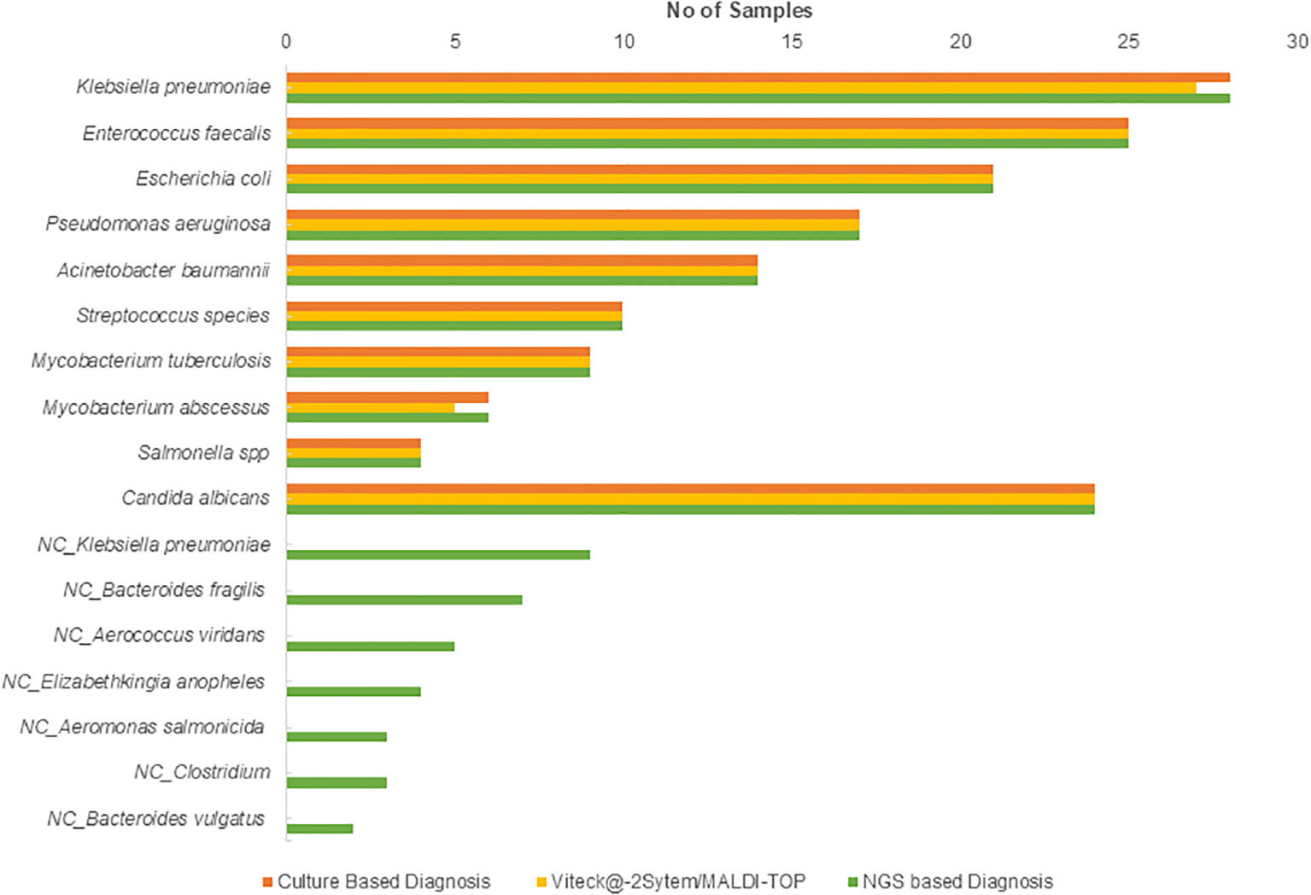
Distribution of bacterial and fungal pathogens identified by traditional culture, MALDI-TOF, and VITEK^@-^2 system. NC, non-cultured.

Out of the 500 collected blood samples, 26.8% (n=134) showed bacterial growth via traditional culture methods, while 4.8% (n=24) were positive for fungal growth (**Figure 1**). The distribution of identified bacterial pathogens in culture-positive samples included *Klebsiella pneumoniae* 20.89% (n=28), *Enterococcus faecalis* 18.65% (n=25), *Escherichia coli* 15.67% (n=21), *Pseudomonas aeruginosa* 12.68% (n=17), *Acinetobacter baumannii* 10.44% (n=14), various *Streptococcus* species 7.46% (n=10), *Mycobacterium tuberculosis* 6.71% (n=9), *Mycobacterium abscessus* 4.47% (n=6), and *Salmonella spp* 2.98% (n=4). Both MALDI-TOF and VITEK^@^2 systems identified bacterial growth in 26.4% (n=132) of specimens, demonstrating comparable results to traditional methods. The distribution of bacterial pathogens identified by MALDI-TOF and VITEK^@^-2 sytem included *Klebsiella pneumoniae* 20.45% (n=27), *Enterococcus faecalis* 18.93% (n=25), *Escherichia coli* 15.90% (n=21), *Pseudomonas aeruginosa* 12.87% (n=17), *Acinetobacter baumannii* 10.60% (n=14), various *Streptococcus* species 7.75% (n=10), *Mycobacterium tuberculosis* 6.8% (n=9), *Mycobacterium abscessus* 3.78% (n=5), and *Salmonella spp* 3% (n=4). *Candida albicans* was reported in 5% (n=24) of samples by traditional culture methods. The results from the MALDI-TOF and VITEK^®^2 compact system were consistent with traditional culture, demonstrating their reliability and effectiveness in identifying bloodstream pathogens.

### NGS based Identification

NGS-based Bactfast detected bacterial presence in 38.2% (n=191) of samples, surpassing traditional culture methods by identifying non-culturable bacteria that were missed. Additionally, non-culture-based NGS identified additional pathogens (n=33) not detected by traditional methods, including *Klebsiella pneumoniae* 27.27% (n=9), *Bacteroides fragilis* 21.21% (n=7), *Aerococcus viridans* 15.15% (n=5), *Elizabethkingia anopheles* 12.12% (n=4), *Aeromonas salmonicida* 9% (n=3), *Clostridium* 9% (n=3), and *Bacteroides vulgatus* 6% (n=2). NGS-based Fungifast showed concordant fungal detection rates with culture methods, identifying *Candida albicans* in 5% (n=24) of samples, consistent with traditional culture results. These additional pathogens detected by NGS highlight its capability to broaden the spectrum of identifiable microorganisms beyond what is achievable through conventional culture methods.

### Traditional vs NGS based diagnosis

A comparison of pathogen detection between traditional culture methods and NGS-based Bactfast revealed significant differences in detection rates (**Table 2**). Notably, NGS-based Bactfast identified non-culturable bacteria missed by traditional methods, highlighting its capability to enhance pathogen detection in clinical samples.

**Table 2:** Comparison of pathogen detection between traditional culture and NGS-based Bactfast and Fungfast.

Pathogen	Traditional Culture (%)	NGS-Based Bactfast (%)	p-value
*Klebsiella pneumoniae*	20.89 (n=28)	7 (n=9)	<0.05
*Enterococcus faecalis*	18.65 (n=25)	–	
*Escherichia coli*	15.67 (n=21)	–	
*Pseudomonas aeruginosa*	12.68 (n=17)	–	
*Acinetobacter baumannii*	10.44 (n=14)	–	
*Various Streptococcus spp*	7.46 (n=10)	–	
*Mycobacterium tuberculosis*	6.71 (n=9)	–	
*Mycobacterium abscessus*	4.47 (n=6)	–	
*Salmonella spp*	2.98 (n=4)	–	
*Candida albicans*	5 (n=24)	5 (n=24)	>0.05
*Bacteroides fragilis*	–	5 (n=7)	
*Aerococcus viridans*	–	4 (n=5)	
*Elizabethkingia anopheles*	–	3 (n=4)	
*Aeromonas salmonicida*	–	2 (n=3)	
*Clostridium*	–	2 (n=3)	
*Bacteroides vulgatus*	–	1 (n=2)	

## Discussion

In the realm of ICUs, where rapid and accurate infectious agent diagnosis is crucial for patient management, NGS technologies have emerged as pivotal tools (Blot et al., 2022). Unlike traditional culture methods, which are limited by their sensitivity and lengthy turnaround times, NGS offers the advantage of detecting a broad range of pathogens directly from clinical samples with unprecedented speed and precision (Peri et al., 2022). This capability is particularly advantageous in ICUs, where infections such as sepsis can escalate rapidly, necessitating prompt intervention (Kumar et al., 2024). The present study aimed to evaluate the clinical utility of NGS-based assays, specifically Bactfast and Fungifast, in an ICU setting. We sought to validate their efficacy in diagnosing bacterial and fungal infections promptly, thereby addressing the critical need for timely therapeutic decisions in critically ill patients. By focusing on a cohort of 500 ICU patients, we investigated the prevalence of bloodstream infections across different clinical conditions with demographic data. In our study, we observed a male predominance among ICU patients (56.4%), reflecting trends seen globally in ICU admissions. Clinical conditions such as sepsis (45%), pneumonia (20%), and urinary tract infections (15%) were predominant, aligning with global patterns of ICU-associated infections (Garland et al., 2018; Merdji et al., 2023). This demographic and clinical distribution underscored the relevance of our study in a broader international context, emphasizing the need for effective diagnostic strategies tailored to regional epidemiological profiles. We have reported comparable detection between the cultural methods vs VITEK^®^2 compact system and MALDI-TOF. The MALDI-TOF is a highly sensitive method so only two pathogens were not detected compared to the culture method and similar reports reported by many other studies. In our study, Bactfast successfully detected bacterial DNA in 38.2% (n=191/500) of blood specimens collected from ICU patients. This outperformed traditional culture methods, which identified bacterial growth in 26.8% (134/500) of samples. We have reported bloodstream infection bacteria including *Klebsiella pneumoniae*, *Enterococcus faecalis*, *Escherichia coli*, *Pseudomonas aeruginosa*, *Acinetobacter baumannii*, various *Streptococcus* species, *Mycobacterium tuberculosis*, *Mycobacterium abscessus*, and *Salmonella* spp. these findings are comparable with the others (Della-Latta et al., 2011). This discrepancy highlights the enhanced sensitivity of NGS technologies like Bactfast in identifying microbial pathogens that may be missed by conventional cultures (Samuel., 2023). Comparative analyses with other studies globally and regionally have consistently shown that culture-based methods typically achieve sensitivities ranging from 60% to 80%, depending on the specific pathogen and clinical context. In contrast, our study demonstrated a higher sensitivity of Bactfast, particularly in detecting anaerobic and fastidious organisms that are challenging to culture (Mahboubi et al., 2016; Schoonbroodt et al., 2022). For instance, studies comparing NGS with traditional methods have revealed that while culture may miss up to 40% of infections due to its limitations in detecting certain microbial species, NGS can achieve near-complete coverage of microbial diversity within a sample. Our findings align with these observations, showing that Bactfast not only identified a broader spectrum of pathogens but also provided rapid results crucial for timely clinical interventions in ICU settings (Rimoldi et al., 2023). Moreover, our study underscores the clinical significance of detecting non-culturable bacteria, which are feasible only through NGS technologies. This capability enhances diagnostic accuracy and informs targeted therapies more effectively compared to traditional approaches. By advocating for the integration of NGS-based assays like Bactfast into routine ICU practices, our research supports the paradigm shift towards more precise and comprehensive infection management strategies. These advanced molecular technologies offer substantial advantages over traditional methods, enabling clinicians to make informed decisions that improve patient outcomes and optimize resource utilization. Our findings may not fully generalize to all ICU settings globally due to variations in patient demographics, clinical conditions, and microbial profiles. Moreover, methodological differences in sample processing, DNA extraction, and bioinformatics pipelines can influence the accuracy and reproducibility of NGS results. The potential for false positives or negatives in NGS results, particularly concerning non-culturable bacteria, is a known limitation. NGS technology can sometimes detect bacterial DNA from non-viable or non-culturable bacteria, leading to false positives (Tan et al., 2015). Conversely, low-abundance pathogens might be missed, resulting in false negatives. Addressing these limitations requires careful interpretation of NGS data and, where possible, corroboration with traditional culture methods (Choi et al., 2021). Standardization across laboratories and platforms is crucial to mitigate these variations. Additionally, the cost and resource-intensive nature of NGS technologies pose barriers to widespread adoption, particularly in resource-limited settings where traditional culture methods remain prevalent due to lower costs and accessibility. Furthermore, while NGS offers high sensitivity in detecting a wide range of pathogens, careful clinical correlation is essential to differentiate between true infection and colonization. Addressing these limitations through collaborative research efforts and technological advancements will be pivotal in enhancing the reliability and clinical utility of NGS-based approaches in ICU infection management.

## Conclusion

In conclusion, our study substantiates the clinical value of Bactfast and Fungifast assays as advanced diagnostic tools in ICU settings. The high sensitivity and rapid detection capabilities of Bactfast for bacterial pathogens demonstrate its potential to complement or even replace conventional culture methods. This is particularly beneficial in scenarios where culture-based approaches may yield inconclusive or delayed results. However, further refinement and larger-scale validation studies are necessary, especially for Fungifast, to establish its sensitivity and specificity in detecting fungal infections accurately. Moving forward, integrating NGS-based assays like Bactfast and Fungifast into routine clinical practice holds promise for improving infection management strategies in ICUs. The ability to quickly identify a broad spectrum of pathogens directly from clinical samples can empower clinicians to make informed treatment decisions promptly, ultimately enhancing patient care and outcomes.

## Data Availability

The original contributions presented in the study are included in the article/supplementary material. Further inquiries can be directed to the corresponding author.
