# Corrigendum: Comparing NGS-based identification of bloodstream infections to traditional culture methods for enhanced ICU care: a comprehensive study

**DOI:** 10.3389/fcimb.2025.1553275

**Published:** 2025-03-27

**Authors:** Wei Wang, Varun Chauhan, Yutian Luo, Sonu Sharma, Chenxi Li, Huaisheng Chen

**Affiliations:** ^1^ Department of Endocrinology, Shenzhen People’s Hospital, Second Clinical Medical College of Jinan University, First Affiliated Hospital of Southern University of Science and Technology, Shenzhen, Guangdong, China; ^2^ Department of Microbiology, Faculty of Applied Sciences and Biotechnology, Shoolini University, Solan, India; ^3^ Department of Critical Care Medicine, Shenzhen People’s Hospital, Second Clinical Medical College of Jinan University, First Affiliated Hospital of Southern University of Science and Technology, Shenzhen, Guangdong, China; ^4^ Department of Pharmacy, DIT University, Mussoorie, Uttarakhand, India

**Keywords:** NGS-based identification in bloodstream infection next-generation sequencing (NGS), bloodstream infections (BSIs), molecular diagnostics, ICU pathogen identification, Bactfast and Fungifast

In the published article, there was an error in Affiliation 2. Instead of “^2^Department of Microbiology, Post-Graduate Institute of Medical Education, Chandigarh, India,”, it should be “Department of Microbiology, Faculty of Applied Sciences and Biotechnology, Shoolini University, Solan, India”.

In the published article, there was an error.

A correction has been made to the **Methods**, 1^st^ Paragraph. This sentence previously stated:

“This study was conducted per the guidelines of the Declaration of Helsinki and approved by the Institutional Ethics Committee of the Post-Graduate Institute of Medical Education with reference number: 2022-EC-025 which complies with international ethical standards. All experiments were compliant with the hospital biosafety regulations.”

The corrected sentence appears below:

“This study was conducted per the guidelines of the Declaration of Helsinki and approved by the Institutional Ethics Committee of the Shoolini University with reference number: SUBMS-2022-EC-025 which complies with international ethical standards. All experiments were compliant with the institutional biosafety regulations.”

A correction has been made to the **Abstract**, 1^st^ line. This sentence previously stated:

“We collected 500 non-duplicate blood samples from ICU patients between January 2023 and December 2023.”

The corrected sentence appears below:

“We collected 500 non-duplicate blood samples from ICU patients between May 2019 and May 2020.”

A correction has been made to **Methods**, *Study design and sample collection*, 1^st^ Paragraph. This sentence previously stated:

“This is the single centre study conducted between Jan 2023 to Dec 2023 at Department of the Microbiology.”

The corrected sentence appears below:

“This is the single centre study conducted between May 2019 to May 2020 at Department of the Microbiology, in Shoolini University.”

A correction has been made to **Ethics statement**,

This sentence previously stated:

“This study was conducted per the guidelines of the Declaration of Helsinki and approved by the Institutional Ethics Committee of the Post-Graduate Institute of Medical Education with reference number: 2022-EC-025 which complies with international ethical standards…”

The corrected sentence appears below:

“This study was conducted per the guidelines of the Declaration of Helsinki and approved by the Institutional Ethics Committee of the Shoolini University with reference number: SUBMS-2022-EC-025 which complies with international ethical standards.”

The authors apologize for this error and state that this does not change the scientific conclusions of the article in any way. The original article has been updated.

